# Impact of the COVID-19 pandemic and associated non-pharmaceutical interventions on other notifiable infectious diseases in Germany: An analysis of national surveillance data during week 1–2016 – week 32–2020

**DOI:** 10.1016/j.lanepe.2021.100103

**Published:** 2021-06-19

**Authors:** Alexander Ullrich, Madlen Schranz, Ute Rexroth, Osamah Hamouda, Lars Schaade, Michaela Diercke, T. Sonia Boender, Robert Koch's Infectious Disease Surveillance Group

**Affiliations:** a Robert Koch Institute, Department for Infectious Disease Epidemiology, Seestrasse 10, 13353 Berlin, Germany; b Robert Koch Institute, Centre for Biological Threats and Special Pathogens, Nordufer 20, 13353 Berlin, Germany

**Keywords:** Public health surveillance, COVID-19, Pandemics, Disease transmission, infectious, Cohort studies, General Practitioners, Epidemiology

## Abstract

**Background:**

The COVID-19 pandemic and associated non-pharmaceutical interventions (NPIs) affect healthcare seeking behaviour, access to healthcare, test strategies, disease notification and workload at public health authorities, but may also lead to a true change in transmission dynamics. We aimed to assess the impact of the pandemic and NPIs on other notifiable infectious diseases under surveillance in Germany.

**Methods:**

We included 32 nationally notifiable disease categories with case numbers >100/year in 2016–2019. We used quasi-Poisson regression analysis on a weekly aggregated time-series incorporating trend and seasonality, to compute the relative change in case numbers during week 2020–10 to 2020–32 (pandemic/NPIs), in comparison to week 2016–01 to 2020–09.

**Findings:**

During week 2020–10 to 2020–32, 216,825 COVID-19 cases, and 162,942 (-35%) cases of other diseases, were notified. Case numbers decreased across all ages and notification categories (all *p*<0•05), except for tick-borne encephalitis, which increased (+58%). The number of cases decreased most for respiratory diseases (from -86% for measles, to -12% for tuberculosis), gastro-intestinal diseases (from -83% for rotavirus gastroenteritis, to -7% for yersiniosis) and imported vector-borne diseases (-75% dengue fever, -73% malaria). The less affected infections were healthcare associated pathogens (from -43% infection/colonisation with carbapenem-non-susceptible *Acinetobacter*, to -28% for Methicillin-resistant *Staphylococcus aureus* invasive infection) and sexually transmitted and blood-borne diseases (from -28% for hepatitis B, to -12% for syphilis).

**Interpretation:**

During the COVID-19 pandemic a drastic decrease of notifications for most infectious diseases and pathogens was observed. Our findings suggest effects of NPIs on overall disease transmission that require further investigation.

**Funding:**

The Robert Koch Institute is the National Public Health Institute of Germany, and is an institute within the portfolio of the Federal Ministry of Health.

**Research in context**

**Evidence before this study**

We conducted a PubMed search for titles and abstracts including the search terms ("COVID-19″ [Supplementary Concept]) AND "Population Surveillance"[Mesh] AND "prevention and control" [Subheading] as well as "non-pharmaceutical intervention*" from database inception to 19 November 2020. In addition, we reviewed the references of relevant studies. Studies on the impact of the COVID-19 pandemic and its associated non-pharmaceutical interventions (NPIs) on routine infectious disease surveillance were scarce and mostly limited to influenza and other respiratory illnesses during the first wave of COVID-19. Typically, public health research on NPIs focused solely on their impact on COVID-19 transmission. However, a decrease in healthcare utilisation during the first months of the pandemic has been documented in different settings (emergency department visits, hospital admissions, primary care) and for a number of non-communicable diseases (including cardiovascular disease, psychiatric emergencies).

**Added value of this study**

To our knowledge, this study is the first to examine the impact of the COVID-19 pandemic and associated NPIs on the full spectrum of infectious diseases under national surveillance. We showed a significant decrease of notifications for almost all notifiable diseases during the COVID-19 pandemic compared to previous years, taking seasonality and trends over time into account. The reason for this changing dynamic is multifactorial, including a reduction in healthcare utilisation, and reduced transmission due to NPIs and changes in mobility.

**Implications of all the available evidence**

The changes in health care utilisation behaviour as well as decreasing cases of infectious and non-communicable diseases show the massive impact that the COVID-19 pandemic and its associated NPIs had, and continues to have, on our societies and health systems worldwide. Surveillance systems face the challenge of dealing with variances in case numbers and identifying appropriate references for all infectious diseases, under these unprecedented circumstances. Despite the dominance of COVID-19 surveillance, other infectious disease must not be overlooked. Changes in transmission patterns but also underdiagnosis and changes in healthcare utilisation behaviour must be monitored closely and call for alignment of public health measures. Vigilant and consistent infectious disease surveillance as well as further research is required to better understand the reasons behind those causal dynamics and to anticipate structural changes affecting routine surveillance.

Alt-text: Unlabelled box

## 1 Introduction

In response to the Coronavirus disease 2019 (COVID-19) pandemic, public health authorities and healthcare providers have had to prioritise their work towards COVID-19 management, since the beginning of 2020. During peak incidence of COVID-19 cases, healthcare providers have often deprioritised elective healthcare [1], or switched to virtual consultations if possible. In addition, healthcare seeking behaviour has changed drastically, as shown by drops in GP and emergency department attendances [2], and fewer hospital admissions for acute coronary syndrome [3]. These changes in healthcare seeking behaviour are likely to be multifactorial, and could potentially be due to reluctance to attend health-facilities because of concerns of catching COVID-19. In Germany, following the initial containment of a cluster of COVID-19 cases starting at the end of January 2020 [4], sustained transmission of SARS-CoV-2 commenced end of February 2020, followed by implementation of the first national public health measures (i.e. non-pharmaceutical interventions, NPIs) in March 2020, as described with more detail in the **Box**.

**Box. Timeline of the COVID-19 epidemic and associated non-pharmaceutical interventions in Germany, January-August 2020**

In Germany, following the initial containment of a cluster of COVID-19 cases starting at the end of January 2020 [4], community transmission of SARS-CoV-2 commenced end of February 2020, following the introductions of cases from Italy and Austria (often related to people returning from ski holidays), and clusters of cases associated with carnival celebrations and other mass gathering events [37]. On 10 March 2020 (week 11-2020), initial measures (i.e. non-pharmaceutical interventions, NPIs) banning mass gatherings started, followed by gradual closing of schools and day care facilities, and a world-wide non-essential travel ban. On 23 March 2020 (week 13-2020), strict physical distancing measures were put in place on a national level (“lockdown”): contact with one other person outside of one's household was allowed. Shops, restaurants, hairdressers and other salons requiring close physical contact were closed; being outside for grocery shopping, medical appointments and sports was permitted. In addition, international travel restrictions and quarantine measures for those entering Germany were put in place for those traveling from areas with high COVID-19 rates. On 20 April 2020, the first loosening of measures started, by allowing shops of certain sizes to reopen, followed by allowing for larger (though restricted) numbers of people to gather. Different levels of contact restrictions, promotion of hand-hygiene, and the use of face masks in closed (public) spaces such as public transport, stores and the workplace, remained in place till the end of the observational period (week 32-2020) [38].

Alt-text: Unlabelled box

Research on the impact of the COVID-19 pandemic and associated NPIs on the (public) health system have largely focussed on COVID-19 transmission (**Research in Context**). However, changes in case notifications of other infectious diseases under surveillance have been observed, particularly for respiratory infections [5] and seasonal influenza [6], [7], [8], [9], but also for measles [10]. These changes could have been related to differences in healthcare seeking behaviour, access to healthcare, test strategies, alternations in disease notification and workload at public health authorities, but may be also driven by a true change in transmission dynamics, initiated by the NPIs.

To understand the epidemiology of all infectious diseases under surveillance during the pandemic, we aimed to assess the impact of the COVID-19 pandemic and NPIs on notifiable infectious diseases under surveillance in Germany.

## 2 Methods

### 2.1 Design & setting

We analysed data from the reporting system for surveillance of notifiable infectious diseases in Germany [11, 12]. As of 1 January 2001, the Protection Against Infection Act determines which infectious diseases (§6, notifications by medical doctors) and which detected pathogens (§7, notifications by laboratories) are notifiable [13]. Case definitions define epidemiological, clinical and laboratory criteria for each notifiable disease [14]; COVID-19 was added as a notifiable disease in Germany on 1 February 2020.

Clinicians and laboratories report cases of infectious diseases and pathogen detection to local health authorities in 412 counties, from where they are forwarded via the state public health authority to the Robert Koch Institute (RKI, national public health institute), except for the detection of HIV, *Treponema pallidum* (syphilis) and *Plasmodium* spp. (malaria) which are reported by laboratories directly to RKI. The RKI provides an overview of the epidemiology of all notifiable diseases in Germany on a regular basis [15].

### 2.2 Data source & inclusion criteria

Case information was retrieved from the notifiable disease database at RKI. We included disease categories that are notifiable in all federal states, with a date of notification between 1 January 2016 (week 01–2016) and 9 August 2020 (week 32–2020). We excluded disease categories with <100 cases per year in 2016–2019 (i.e. Zika virus disease, Chikungunya virus disease, Leptospirosis, Rubella, Brucellosis and Tularaemia), because the planned statistical analysis by week was not suitable for small case numbers, nor was it of interest to study the relationship between the pandemic, NPIs and dynamics on relatively rare diseases. Additionally, hantavirus disease, adenovirus conjunctivitis and invasive pneumococcal diseases were excluded due to complex epidemiological dynamics, changes in case definitions, or very limited time under surveillance. Included and excluded disease categories and their reason for exclusion are summarised in **Supplemental Table 1**.

### 2.3 Variables & definitions

For each notification, we extracted the date of notification, date of disease onset, age, sex, county, state notification category, and case definition from the notifiable disease database as of 8 November 2020.

We grouped the included notification categories into five main groups: One, respiratory diseases (including vaccine-preventable and childhood-related diseases), including chickenpox, invasive *Haemophilus influenzae* infection, seasonal influenza, legionellosis, measles, invasive meningococcal disease, mumps, whooping cough, and tuberculosis. Two, gastro-intestinal diseases, including, *Campylobacter*-enteritis, cryptosporidiosis, enterohemorrhagic *Escherichia coli* (EHEC)-disease, giardiasis, hepatitis A, hepatitis E, listeriosis, norovirus gastroenteritis, rotavirus gastroenteritis, salmonellosis, shigellosis, yersiniosis. Three, cases of healthcare associated pathogens, including *Clostridioides difficile* infections with a severe clinical course, infection or colonisation with carbapenem-non-susceptible *Acinetobacter* (CRA) and Enterobacterales (CRE), and invasive infection with Methicillin-resistant *Staphylococcus aureus* (MRSA). Data for the disease categories *C. difficile*, CRA and CRE were only available from 1 January 2017 onwards. Fourth, sexually transmitted and blood-borne diseases, including hepatitis B, and C, HIV infection, and syphilis. For hepatitis B and C, cases were included from 1 January 2018 onwards, due to changes in case definitions. Fifth and last, vector-borne diseases (endemic & imported), including tick-borne encephalitis (TBE), dengue fever, and malaria.

### 2.4 Ethics statement

Pseudonymized notification data was collected at the RKI based on the German Infection Protection Act [13].

### 2.5 Statistical methods

We assessed the impact of the COVID-19 pandemic and associated NPIs on case notification of other notifiable infectious diseases using two statistical approaches described in detail below. First, we calculated the overall and age-stratified change in case notifications for all notifications combined (except for COVID-19) for the weeks 10 to 32 in 2020, compared to the same weeks in 2016–2019. Second, we calculated disease-specific estimates based on weekly aggregated times-series using the full observation period.

First, we determined the overall change in notification numbers amongst all notification categories (except COVID-19) during the reporting week 10–32 for the years 2016 to 2020 using negative binomial regression. The regression model included notification numbers (count) and a binary pandemic variable, which was set to 1 for the pandemic year (2020) and 0 for all other years (2016, 2017, 2018, 2019); see Eq. (1). There was no apparent trend amongst overall notification numbers. Seasonality was implicitly modelled through the data selection on a specific time period of the year, i.e. week 10–32. In practice, the exponential of the regression coefficient of the binary pandemic variable represented the change in notification numbers during the pandemic year 2020, compared to the numbers in previous years. I.e., the exponential of the regression coefficient (exp($\beta_{1}$)) of 0.65 meant that notification numbers were only at a level of 65% of the expected numbers based on previous years, in turn meaning a 35% decrease in notifications. Confidence intervals for the regression coefficient were derived by profiling of the likelihood function, the 95% confidence interval (95%CI) was reported. The same analysis was performed for each of the six age groups 0–4 years, 5–14 years, 15–34 years, 35–59 years, 60–79 years and 80+ years.

(1) $$\log\left(E\left(count\right)\right)=\alpha +\beta_{1}\left(pandemic\right)$$

Second, to determine the relative change in notification numbers for each specific notification category, we used a quasi-Poisson regression analysis on a weekly aggregated time-series (date of notification). This regression model (Eq. (2)) incorporated variables for trend, seasonality, and a binary pandemic variable (analogous to the model above: 1 for pandemic reporting weeks 2020–10 to 2020–32, and 0 for all other weeks, i.e. 2016–01 to 2020–09). From the exponential of the regression coefficient ($\beta_{1})$ for the binary pandemic variable, we derived the relative difference between the expected number of notifications based on notifications in previous years and the actual number of notifications during the COVID-19 pandemic (week 2020–10 to 2020–32). The relative change per week was computed based on the difference between the actual number of notifications for one week and the expected value for the same week, under the assumption that no pandemic or NPIs were present (week 2016–01 to 2020–09). This expected value, the difference, and 95%CI, were computed for the weeks from 2020 to 01 to 2020–32.

(2) $$\log\left(E\left(count\right)\right)=\alpha +\beta_{1}\left(pandemic\right)+\beta_{2}\left(trend\right)+\beta_{3}\left(\sin\left(2\pi *\;week/52\right)+\cos\left(2\pi *\;week/52\right)\right)$$

In addition, we investigated whether the overall change (in %) in notification numbers amongst all other disease categories was associated with the COVID-19 case load. To this end, we computed the overall change in notifications (all disease categories except COVID-19) and the number of COVID-19 cases (absolute, and as cases per 100,000 inhabitants) for each of the 412 counties. We computed the Pearson correlation and used the *t-test* statistic to test for a linear relationship between the overall relative change and the COVID-19 incidence, the number of cases and the population, respectively. Correlations were considered statistically significant, when *p* < 0.05. All data analysis was performed in R (Version 3.6.1); using the *dplyr, tidyr, MASS, stats, ggstatsplot, ggplot2* packages.

### 2.6 Role of the funding source

The Robert Koch Institute is the National Public Health Institute of Germany, and is an institute within the portfolio of the Federal Ministry of Health. The funders had no role in study design, data collection, data analysis, interpretation, writing of the report.

## 3 Results

### 3.1 All notifiable diseases in Germany, including COVID-19

We included 32 nationally notifiable disease categories that had reported on average >100 cases per year before the COVID-19 pandemic and NPIs started (**Supplemental Figure 1**). Between week 10 and 32 in 2020, 379,767 cases were reported to RKI, of whom the majority (*n* = 216,825; 57%) were COVID-19 cases. During the same period, 162,942 cases of all reported infectious diseases (except COVID-19) were recorded, which was −35% (95%CI: −58%; −2%) compared to previous years same time period (2016: 219,752; 2017: 195,776; 2018: 344,636; 2019: 247,692), irrespective of disease category. There was no statistically significant correlation between the county-level COVID-19 incidence and the relative change in number of cases for all other infectious diseases. Neither the COVID-19 case load or incidence, nor population size of the county was associated with the overall decrease in case numbers (**Supplemental Figure 2**).

**Fig. 1:**
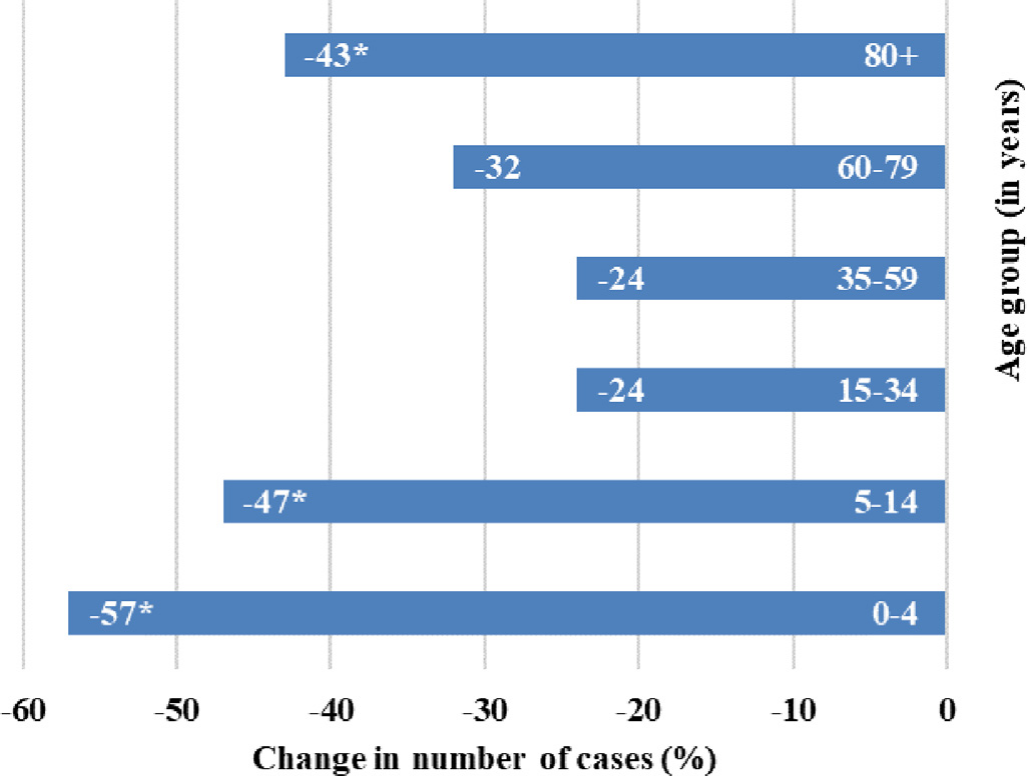
Relative change (%) in the number of cases in different age groups during the first months of the COVID-19 pandemic and non-pharmaceutical interventions, compared to the expected number of cases. * Statistically significant changes (*p*<0.05): 0–4 years −57% (95%CI: −66%; −45%); 5–14 years −47% (95%CI: −61%; −29%), 15–34 years −24% (95%CI: −45%; 4%), 35–59 years −24% (95%CI: −59%; 41%), 60–79 years −32% (95%CI: −61%; 19%), 80+ years −43% (95%CI: −64%, −9%).

Case numbers seemed to decrease across all ages, with the largest and statistically significant decreases observed amongst those under 14 years of age (0–4 years −57% [95%CI: −66%; −45%]; 5–14 years −47% [95%CI: −61%; −29%]) and over 80 years of age (−43% [95%CI: −64%, −9%]) (Fig. 1). No difference in overall change in the number of cases were found by sex. All disease categories changed significantly compared to previous years (p-value < 0.05) (Table 1). Case numbers decreased across all notification categories ranging from −83% to −7%, except for TBE, which increased +58%.

**Table 1:** National surveillance of infectious diseases in Germany: relative change (∆%) in number of cases in week 10 to 32 in 2020, compared to the expected numbers based on the historical data starting from 2016.

CRE = *Enterobacterales*, carbapenem-non-susceptible; MRSA = Methicillin-resistant *Staphylococcus aureus*. Seasonal influenza is not shown after week 20 (out of season).

No calculation:.

### 3.2 Respiratory diseases

**Fig. 2:**
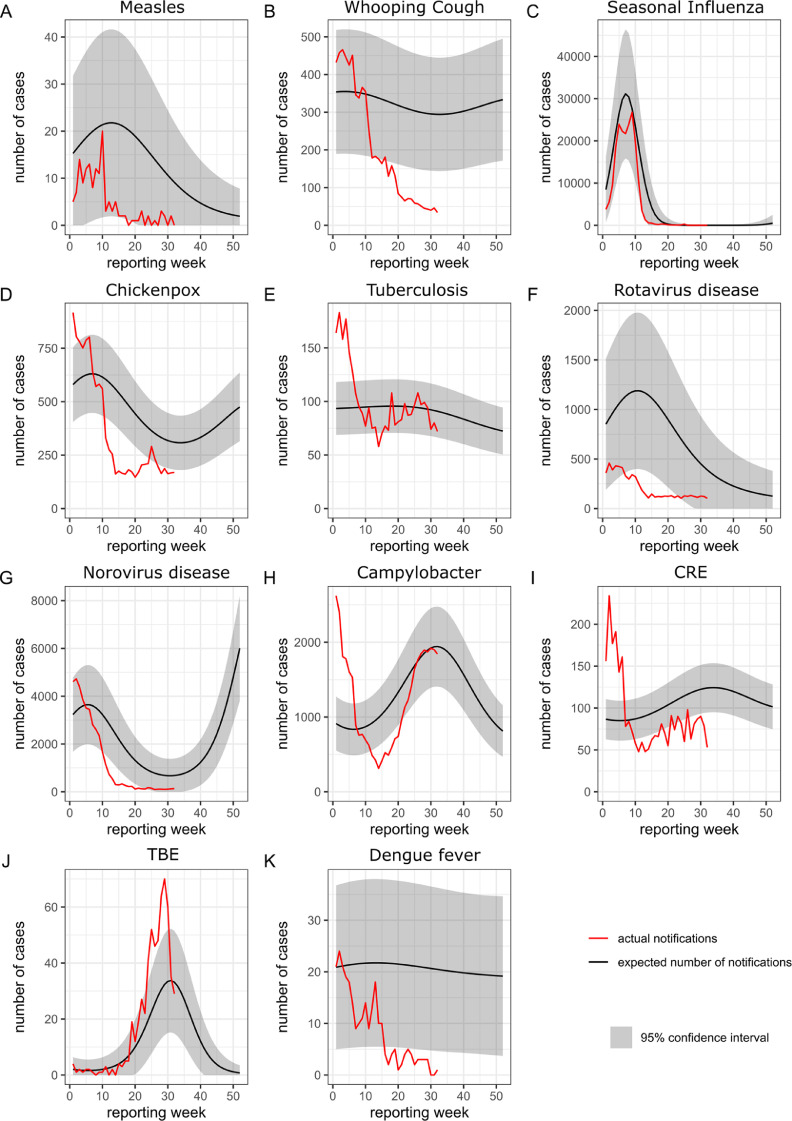
Temporal variation in case numbers notified in week 1 to 32 in 2020, compared to the expected case numbers, of a selection of infectious diseases under surveillance in Germany. CRE = Enterobacterales, carbapenem-non-susceptible, TBE = tick-borne encephalitis. The number of expected cases was estimated with quasi-Poisson regression analysis, using case data of week 2016–01 to 2020–09. The regression model incorporated trend and seasonal components.

All included respiratory diseases showed a substantial and statistically significant reduction of cases during the first months of the pandemic and NPIs (range: −85•5% to −11•6%), compared to the expected number of cases. The largest decrease was observed for measles, which decreased by −85•5% (95%CI: −89•0%; −81•0%, Fig. 2**A**). Whooping cough also showed a large decrease in cases (−63•7% [95%CI: −65•2%; −62•2%], Fig. 2**B**), followed by invasive *Haemophilus influenzae* infection (−61.3% [95%CI: −67•4%; −54•2%]), seasonal influenza (−54•4% [95%CI: −54•9%; −53•9%], Fig. 2**C**), chickenpox (−51•5% [95%CI: −53•0%; −50•0%], Fig. 2**D**), invasive meningococcal disease (−47•1% [95%CI: −55•2%; −37•6%]), and mumps (−33•3% [95%CI: −42•8%; −22•1%]). Legionellosis cases decreased by −27•8% (95%CI: −34•2; −20•7), and tuberculosis cases by −11•6% (95%CI: −11•6; −6•6, Fig. 2**E**).

### 3.3 Gastro-intestinal diseases

All included gastro-intestinal diseases showed significant reductions in cases (range: −83•3% to −7•0%), compared to the expected number of cases based on previous years. The largest decreases were seen amongst rotavirus disease (−83•3% [95%CI: −83•9%; −82•7%], Fig. 2**F**), shigellosis (−82•9% [95%CI: −87•0%; −77•6%]), and norovirus disease (−78•7% [95%CI: −79•2%; −78•2%], Fig. 2**G**). Case numbers of cryptosporidiosis (−52•4% [95%CI: −57•2%; −47•0]), EHEC-disease (−46•4% [95%CI: −50•9%; −41•5%]), salmonellosis (−45•5% [95%CI: −47•4%; −43•4%]), giardiasis (−43•3% [95%CI: −47•3%;−39•0%]), hepatitis A (−36•7% [95%CI: −43•5%; −29•1%]), *Campylobacter*-enteritis (−22•2% [95%CI: −23•4%; −21•0%], Fig. 2**H**), and listeriosis also showed substantial decreases. Hepatitis E (−7•0% [95%CI: −10•9%; −3•0%]) and yersiniosis (−7•0% [95%CI: −13•5%; −0•0%]) were also reported less frequently than expected.

### 3.4 Healthcare associated pathogens

The number of cases of all pathogens in this category showed an overall substantial and statistically significant reduction (range: −42•7% to −28•0) during the first months of the pandemic and NPIs, compared to the expected number of cases based on previous years: CRA −42•7% (95%CI: −52•0%; −31•6%), *C. difficile* infections with a severe clinical course −32•4% (95%CI: −37•5%; −24•6%), CRE −34•6% (95%CI: −38•6%; −30•2%, Fig. 2**I**); and MRSA invasive infection −28•0 (95%CI: −34•6%; −20•7%).

### 3.5 Sexually transmitted and blood-borne diseases

During the first months of the pandemic and NPIs, the expected number of hepatitis B and hepatitis C cases decreased by −28•3% (95%CI: −32•0%; −24•4%) and −27•7% (95%CI: −31•8%; −23•4%) respectively, compared to the expected number based on notification data from 2018 to 2019. HIV cases decreased by −22•1% (95%CI: −27•6%; −16•1%), and syphilis by −12•1% (95%CI: −15•8%; −8•2%), compared to expected number of cases, based on 2016–2019.

### 3.6 Vector-borne diseases

The number of dengue fever cases decreased substantially by −75•1% (95%CI: −79•5%; −69•9%, Fig. 2**K**), compared to the expected number of cases. Likewise, the number of malaria (*Plasmodium* spp.) cases decreased by −73•0 (95%CI: −77•7%; −67•4%).

TBE was the only notification category that showed a significant increase in case numbers during the first months of the pandemic and NPIs, compared to the expected number based on previous years: +57•7% (95%CI: +37•8%; +80•5%, Fig. 2**J**).

## 4 Discussion

During the first months of the COVID-19 pandemic and NPIs in Germany, an extensive change in cases of infectious diseases under routine national surveillance was observed. COVID-19 dominated infectious disease surveillance: more than half of the total reported cases were COVID-19. In parallel, a large decrease in case numbers for nearly all infectious diseases was recorded, especially amongst younger and older age groups, except for TBE, for which we observed a notable increase in case numbers.

The reasons behind this drastic change in notification dynamics are multifactorial, and cannot be fully explained by our analysis of routine surveillance data; the current analysis is subject to limitations summarised here and alongside the disease-group specific discussion. We could not take the perceived health and healthcare seeking behaviour into account, which to a large extent influence subsequent diagnosis and reporting. By close collaboration with all public health specialists responsible for the routine monitoring and surveillance of these pathogens, we aimed to increase our awareness of all possible factors that might influence or bias these findings in this discussion. In general, the notification system depends on diagnosis of infectious diseases by physicians and laboratories. Therefore, health care seeking behaviour and the number of laboratory tests conducted play an important role for capturing a case in the system. Further investigation of the full cascade of care is needed to understand if and to what extent underdiagnosis played a role in the decrease in notifications. The national reference centres for salmonellosis and listeriosis reported a decrease in the number of samples being sent to laboratories, which ruled out potential de-prioritisation or delays because of an increase in samples for SARS-CoV-2 diagnostics at laboratories [16]. We found no significant correlation on county level between reported COVID-19 cases and overall decrease of notification for the investigated diseases. Furthermore, epidemiological factors such as circulating virus strains (e.g. seasonal influenza, rotavirus), multi-annual seasonality (e.g. measles, TBE) and large outbreaks (which can affect both the size of the susceptible population over time, as well as the case numbers), could not be accounted for in the current analysis, and may have introduced bias. In addition, various infectious diseases follow mixed patterns of transmission. For example, hepatitis A can be both food-borne, transmitted from person-to-person, as well as travel-associated. These dynamics cannot be untangled in this study. Moreover, the observation time is too short to monitor dynamics in transmission of pathogens with long incubation times (e.g. tuberculosis, hepatitis B and C, HIV). Of note, we also limited our current analysis to diseases under national surveillance, i.e. excluding diseases that are notifiable in a selection of states such as Lyme disease, chlamydia, and hand, foot, and mouth disease.

NPIs, including school closures, physical distancing, and enhanced hand-hygiene, likely resulted in a reduction in transmission of respiratory diseases included in our analysis. The large drop in measles cases, while being in-season, is striking, and likely to be multicausal. Measles outbreaks in Germany usually start with a travel-associated introduction, followed by outbreaks, which were less likely to occur due to border closure and travel restrictions. A recent analysis of measles surveillance indicated drops in reported case numbers across the EE/EEA and UK till May 2020, which could be due to underdiagnosis, underreporting, but potentially also a reduction in circulation [10]. A reduction in transmission and, to a lesser extent, a potential reduction in presentation to care and underdiagnosis are considered the main reasons for the decrease of measles case numbers. Lack of laboratory capacity and a potential backlog in notifications have been rather ruled out by state health authorities [personal communication: Dorothea Matysiak-Klose, RKI, infectious disease epidemiology, vaccine preventable diseases] and the national reference centre [personal communication: Annette Mankertz, RKI, national reference centre for measles, mumps, rubella]. In addition to measles, the NPIs physical distancing, school and day-care closures, and face masque wearing, have reduced the likelihood of transmission of many vaccine-preventable and childhood associated infections with droplet/airborne transmission, including pertussis, invasive *Haemophilus influenzae* infection, chickenpox, invasive meningococcal disease, and mumps.

The impact of the first months of the COVID-19 pandemic and NPIs on seasonal influenza during the observation period is multifactorial. First, the 2019/2020 influenza season was assessed to be moderate compared to previous seasons, and was close to its end when the COVID-19 pandemic started [17]. Second, influenza notifications are influenced by testing behaviour for influenza and other respiratory diseases, which in turn were affected by the severity of (current and previous) influenza seasons on the one hand, and by testing behaviour for COVID-19 on the other. Alterations in notification data therefore do not directly translate into a change in the underlying epidemiology. Nonetheless, the influenza season abruptly ended about two weeks earlier than expected, as confirmed by syndromic and virologic surveillance systems for acute respiratory illness [17], [18], [19]. NPIs aiming to prevent transmission of COVID-19 are likely to have prevented transmission of seasonal influenza as well. Decreased influenza activity during the COVID-19 pandemic was reported in several countries in both the northern and southern hemisphere, where its decrease was associated with COVID-19 infection prevention measures [6, 9, 20, 21].

A reduction in case numbers of gastro-intestinal diseases was observed across all age groups. NPIs for COVID-19 including enhanced hand-hygiene, closure of day care facilities and home office work also prevent human-to-human transmission of gastro-intestinal infections. Travel restrictions prevented importation of cases from endemic countries. Even though there was a change in eating habits (i.e. people ate less often in restaurants and more at home), people remained at risk for food-borne infections. This supports the hypothesis that the reduction in case numbers is also related to under-diagnosis, due to a change in healthcare utilisation behaviour [16, 22, 23].

Notifications of the healthcare associated pathogens *C. difficile* infection with a severe clinical course, CRA, CRE, and MRSA decreased by about one third. This reduction might be due to COVID-19 related increased infection prevention and control measures within hospitals. The cancellation and rescheduling of many elective procedures, and a reduction in overall healthcare utilisation, have led to a reduction in absolute patient numbers [1, 2] and therefore a reduction of these infections and pathogens. Antimicrobial resistant pathogens could either increase or decrease during the COVID-19 pandemic, in particular antibiotic prescribing and infection prevention and control practices [24]; the impact of the pandemic on antimicrobial resistance will only become clear in the coming months and years as data gradually become available. In the long run, it is expected that the impact of the COVID-19 pandemic is more likely to increase the development of antimicrobial resistance, due to overuse of antibiotics, wide use of biocidal agents for environmental and personal disinfection, and disruption of health services and treatment [25].

Case numbers of sexually transmitted and blood-borne infections with hepatitis B and C, HIV, and syphilis have all decreased during the first months of the COVID-19 pandemic. Interpretation of this reduction is challenging for several reasons. Caution is warranted when interpreting notification data over time, because of a short reference period, and recent changes in the hepatitis B and C case definitions [26, 27]. Furthermore, case notification of HIV and syphilis follows an ongoing process of laboratory-based anonymous reporting, which is supplemented by clinical data of medical doctors, followed by deduplication on national level. Therefore, the set of HIV and syphilis of case notifications included in this analysis (throughout the course of 2020) might include a slightly different set of cases than the annual data lock presented for yearly reporting [15]. Additionally, diagnosis of these infections often occurs in the chronic stage of disease.

Behaviour plays a large role in sexually transmitted and blood-borne infections. A Dutch survey indicated that singles were dating less, and had less partners during the pandemic, related to contact reduction, and closure of nightclubs and bars [28], which could in turn have led to a reduction in STI transmission. An ad-hoc survey amongst users of the gay dating platform GayRomeo (predominantly from Germany) in early April 2020 showed a dramatic reduction of partner seeking [29], and HIV-PrEP uptake monitoring showed a strong transient reduction of first PrEP uptake visits during the second quarter of 2020. Conversely, the reduction in notifications could also be a result of a reduction in healthcare seeking behaviour. Importantly, de-prioritisation and interruptions of sexual health service provision has challenged access to testing facilities [30]. A recent survey amongst German Hepatitis C-Registry centers showed a decrease of consultations of hepatitis C patients and delayed diagnoses during lockdown regulations in the first COVID-19 wave, which may support this finding [31]. Local health authorities often had to de-prioritise these services due to a focus on the pandemic response. Furthermore, the closure of low-threshold facilities has put injecting drug users at higher risk of infection [32], which is of concern. Without adequate service provisions, sexually transmitted and blood-borne infections can remain undetected, and might in turn lead to increase in spread.

Travel restrictions and border closure prevented new importation of infectious diseases that are not endemic in Germany, which had a direct effect on dengue fever and malaria notifications. Most cases notified during the early pandemic phase likely resulted from pre-pandemic travel. In addition, travel restrictions have prevented imported cases of gastro-enteritis (e.g. hepatitis A, giardiasis, cryptosporidium). Despite their respiratory transmission routes, the reduction in cases of measles, legionellosis and tuberculosis in Germany are also likely to be due to a reduction in mobility because of travel (measles, legionellosis[33]) and migration (tuberculosis), and less likely a consequence of a reduction in respiratory transmission. Imported measles cases were the main reason for regional outbreaks in recent years [15].

The only increase in case numbers was observed for TBE. The increase was substantial, though likely to be multifactorial. One factor could be increased engagement in outdoor activities in endemic areas in Germany, because other recreational activities were limited due to the public health measures. However, the increase may also be related to high tick counts, as observed in 2020 in several regularly flagged areas, as well as a higher proportion of adult ticks, which have a higher TBE virus prevalence than the younger tick stadia [personal communication: Gerhard Dobler, German TBE consultant laboratory]

The multicausality behind these dynamics in disease notifications cannot be fully explained, and highlights the need for vigilant infectious disease surveillance, during the protracting emergency of COVID-19. At the same time, the use of routine surveillance data is a strength, as this enables long term assessments, comparisons with routine notification data in other systems, and can contribute to future preparedness and response. A true potential reduction in transmission of infectious diseases under these NPIs is reassuring in terms of effectivity of the measures to prevent infectious disease transmission, which reduces the burden of disease in the population, as well as the burden on the healthcare system. However, potential underdiagnosis of infectious diseases is of concern. Future analyses of the data will provide more insight into long-term effects of the pandemic and NPIs on the healthcare system and disease transmission. In addition, future evaluations will be able to indicate whether or not fundamental changes will occur.

Public health communication should highlight safety and accessibility of all preventive and clinical health services, and urge people to seek healthcare when needed. In addition, preventive health services for all infectious diseases, such as vaccination and sexual health services, and low-threshold facilities for injecting drug users, should remain operational as much as possible, and known to be safe and accessible to the public. Importantly, the observed increase in TBE notifications warrants enhanced prevention and control measures adapted to increased outdoor activity, by raising awareness through communication, and vaccination for those at risk, i.e. people living in or visiting TBE endemic areas. Public risk perception of all infectious diseases, and its associated prevention measures, are dynamic and closely monitored to allow swift adaptation to ensure long-term trust in public health authorities [34]. Importantly, the COVID-19 pandemic amplifies socioeconomic inequalities in health, putting those with lower socio-economic status at higher risk for COVID-19, as well as other communicable and non-communicable public health threats [35, 36].

In conclusion, the COVID-19 pandemic and its associated NPIs have drastically affected infectious disease notifications and surveillance, during the first months of the pandemic. Notification data depend on the actual number of infections, healthcare seeking behaviour, diagnosis, and subsequent reporting. The primary public health measures for COVID-19 in Germany, i.e. the NPIs physical distancing, hand and cough hygiene, face masks and ventilation, aim for a reduction in human-to-human transmission of infectious diseases, especially respiratory infections through droplets. The collateral effect of these interventions may have resulted in an overall reduction in transmission for some other infectious diseases. However, caution is warranted because the reduction in notifications does not necessarily mean a reduction in transmission.

## Author Contributors

UR and MD conceived the study. AU designed the initial methodology, which was further developed with MD, MS and TSB. AU conducted the statistical analysis. MS and TSB provided the research in context. TSB wrote the first draft of the manuscript, and aligned the interpretation of the findings with the collaborator group (Robert Koch's Infectious Disease Surveillance Group, listed below), together with all authors. Robert Koch's Infectious Disease Surveillance Group supported the interpretation of specific notification categories, in discussion with TSB. All authors have access to the underlying data, and provided input to, and approved, the final manuscript.

## Robert Koch's Infectious Disease Surveillance Group

In alphabetical order: Bonita Brodhun, Silke Buda, Sandra Dudareva, Tim Eckmanns, Julia Enkelmann, Mirko Faber, Gerhard Falkenhorst, Christina Frank, Barbara Gunsenheimer-Bartmeyer, Wiebke Hellenbrand, Klaus Jansen, Anja Klingeberg, Stefan Kröger, Annette Mankertz, Ulrich Marcus, Dorothea Matysiak-Klose, Annicka Reuss, Bettina Rosner, Julia Schilling, Anette Siedler, Nicole Schmidt, Benedikt Zacher, Ruth Zimmermann.

## Declaration of interests

None of the authors declare competing interests.

## Appendix Supplementary materials

Image, application 1

Image, application 2

Image, image 3

Image, image 4

Image, application 5
