# Supplementary material for: Impact of the COVID-19 pandemic and associated non-pharmaceutical interventions on other notifiable infectious diseases in Germany: An analysis of national surveillance data during week 1–2016 – week 32–2020
Source: Lancet Reg Health Eur. 2021 Jun 19;6:100103. doi: 10.1016/j.lanepe.2021.100103 (PMC8454829; doi:10.1016/j.lanepe.2021.100103)
Supplement: Supplementary file 1 [file mmc1.docx]

**This translation in German was submitted by the authors and we reproduce it as supplied. It has not been peer reviewed. Our editorial processes have only been applied to the original abstract in English, which should serve as reference for this manuscript.**

Einleitung

Die COVID-19-Pandemie und die damit verbundenen nicht-pharmazeutische Interventionen (NPIs) wirken sich auf das Verhalten der Gesundheitssuchenden, den Zugang zur Gesundheitsversorgung, die Teststrategien, die Meldung von Infektionskrankheiten und die Arbeitsbelastung der Gesundheitsbehörden aus, können aber auch zu einer echten Veränderung der Übertragungsdynamik führen. Wir untersuchten die Auswirkungen der COVID-19 Pandemie und damit assoziierter NPIs auf andere meldepflichtige Infektionskrankheiten in Deutschland.

Methoden

Wir schlossen 32 national meldepflichtige Infektionskrankheiten mit >100 Meldungen/Jahr in 2016-2019 ein. Unter Einbeziehung von Trend und Saisonalität berechneten wir mit Hilfe einer quasi-Poisson Regression, basierend auf wöchentlich aggregierten Zeitreihen, die relative Veränderung der Fallzahlen in den Wochen 10/2020 bis 32/2020 (Pandemie und NPIs) im Vergleich zu den Wochen 1/2016 bis 9/2020.

Ergebnisse

In den Wochen 10/2020 bis 32/2020 wurden 216.825 COVID-19-Fälle und 162.942 (-35 %) Fälle anderer Infektionskrankheiten gemeldet. Ein Rückgang der Fallzahlen war über alle Altersgruppen und Meldekategorien hinweg zu beobachten (alle p<0.005). Lediglich für FSME war ein Anstieg zu beobachten (+58%).

Der stärkste Rückgang konnte bei respiratorisch übertragbaren Krankheiten (von -86% für Masern bis -12 % für Tuberkulose), bei gastrointestinalen Krankheiten (von -83% für Rotavirus-Gastroenteritis bis -7% für Yersiniose) und bei importierten vektorübertragbaren Krankheiten (von -75% für Dengue-Fieber bis -73% für Malaria) beobachtet werden. Weniger betroffen waren nosokomiale Infektionen (Rückgang von -43% für Infektion/Kolonisation mit Carbapenem-nicht-empfänglichen *Acinetobacter* bis -28% für invasive Infektion mit Methicillin-resistenten *Staphylococcus aureus)* sowie sexuell und durch Blut übertragbare Krankheiten (von -28 für Hepatitis B bis -12% für Syphilis).

Fazit

Die COVID-19-Pandemie und damit assoziierte NPIs führte bei den meisten Infektionskrankheiten in Deutschland zu einem drastischen Rückgang der Fälle, vor allem bei jüngeren und älteren Altersgruppen. Unsere Ergebnisse deuten auf Effekte von NPIs auf die gesamte Krankheitsübertragung hin, die weiter untersucht werden müssen.

*Sieh auch: Schranz M, Ullrich A, Rexroth U, Hamouda O, Schaade L, Diercke M, Boender S: Die Auswirkungen der COVID-19-Pandemie und assoziierter Public-Health-Maßnahmen auf andere meldepflichtige Infektionskrankheiten in Deutschland (MW 1/2016 – 32/2020) Epid Bull 2021;7:3 -7 |* [*DOI 10.25646/8011*](https://edoc.rki.de/handle/176904/7780)
