# Supplementary material for: Impact of the COVID-19 pandemic and associated non-pharmaceutical interventions on other notifiable infectious diseases in Germany: An analysis of national surveillance data during week 1–2016 – week 32–2020
Source: Lancet Reg Health Eur. 2021 Jun 19;6:100103. doi: 10.1016/j.lanepe.2021.100103 (PMC8454829; doi:10.1016/j.lanepe.2021.100103)
Supplement: Supplementary file 2 [file mmc2.docx]

# Supplemental material

## Supplemental Table 1. Included and excluded disease notification categories, by group.

| Group | Included in this analysis | Excluded from this analysis  (reason for exclusion between brackets) |
| --- | --- | --- |
| Respiratory | Chickenpox;  Invasive *Haemophilus* *influenzae* infection;  Seasonal influenza;  Legionellosis;  Measles;  Invasive meningococcal disease;  Mumps;  Whooping cough;  Tuberculosis | Invasive Pneumococcal disease (notifiable only since March 2020);  Rubella (<100 cases/year); |
| Gastro-intestinal | *Campylobacter* enteritis;  Cryptosporidiosis;  enterohemorrhagic *Escherichia coli* (EHEC)-disease;  Giardiasis;  Hepatitis A;  Hepatitis E;  Listeriosis;  Norovirus gastroenteritis;  Rotavirus gastroenteritis;  Salmonellosis;  Shigellosis;  Yersiniosis | Brucellosis (<100 cases/year);  Leptospirosis (<100 cases/year);  Tularaemia (<100 cases/year) |
| Healthcare associated pathogens | *Clostridioides difficile* infections with a severe clinical course;  infection or colonisation with carbapenem-non-susceptible *Acinetobacter* (CRA);  infection or colonisation with carbapenem-non-susceptible Enterobacterales (CRE),  invasive infection with Methicillin-resistant *Staphylococcus aureus* (MRSA) |  |
| Sexually transmitted and blood-borne | Hepatitis B;  Hepatitis C;  HIV infection;  *Treponema pallidum* (Syphilis) |  |
| Vector-borne | Tick-borne encephalitis;  Dengue fever;  *Plasmodium spp.* (Malaria) | Zika virus disease (<100 cases/year); Chikungunya virus disease (<100 cases/year) |
| Other |  | Adenovirus conjunctivitis;  Hantavirus disease (complex disease dynamics, unsuitable for this analysis) |

Legend: Additionally, hantavirus disease, adenovirus conjunctivitis and invasive pneumococcal diseases were excluded due to complex epidemiological dynamics, changes in case definitions or very limited time under surveillance.

55 disease notification categories under surveillance in Germany, according to the Protection Against Infection Act

14 categories excluded
(only notifiable in a selection of states)

41 disease notification categories under national surveillance in Germany

6 categories (excluded reporting <100 cases/year):
Rubella, Zika virus disease, Brucellosis, Tularemia, and leptospirosis, Chikungunya virus disease

35 disease notification categories under national surveillance in Germany,
reporting >100 cases/year in 2016-2019*

3 additional category exclusions:
Hantavirus, adenovirus conjunctivitis, Pneumococcal disease

32 disease notification categories under national surveillance in Germany, reporting >100 cases/year in 2016-2019*, included in this analysis

## Supplemental Figure 1. Flow chart of inclusion of disease notification categories

* Limited to 2017-2019 for notifications of *Clostridioides difficile* infections with a severe clinical course, infection or colonisation with carbapenem-non-susceptible *Acinetobacter* (CRA) or *Enterobacterales* (CRE); and limited to 2018-2019 for hepatitis B and C notifications.


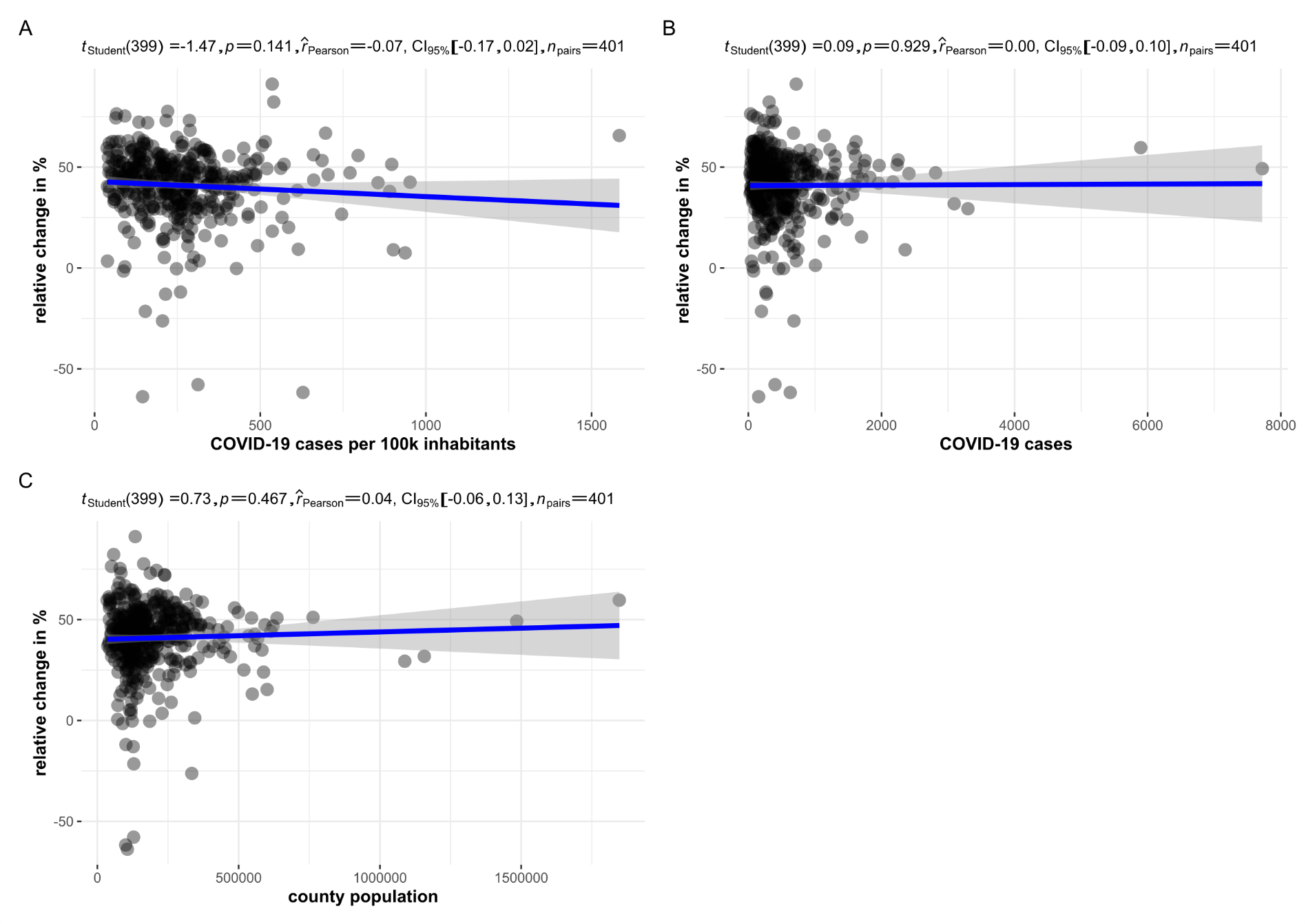


## Supplemental Figure 2.

## Correlation between relative change in number of notifications (all notification categories except COVID-19 cases) with A) COVID-19 incidence B) COVID-19 number of notifications and C) population on county level.

Each dot shows the relative change in notifications and the COVID-19 incidence for one county. The regression line indicates the linear relationship between the change and the COVID-19 incidence, the corresponding p-value, Pearson’s r with 95% confidence interval are reported above.
