# Supplementary figures and images for: Impact of the COVID-19 pandemic and associated non-pharmaceutical interventions on other notifiable infectious diseases in Germany: An analysis of national surveillance data during week 1–2016 – week 32–2020
Source: Lancet Reg Health Eur. 2021 Jun 19;6:100103. doi: 10.1016/j.lanepe.2021.100103 (PMC8454829; doi:10.1016/j.lanepe.2021.100103)

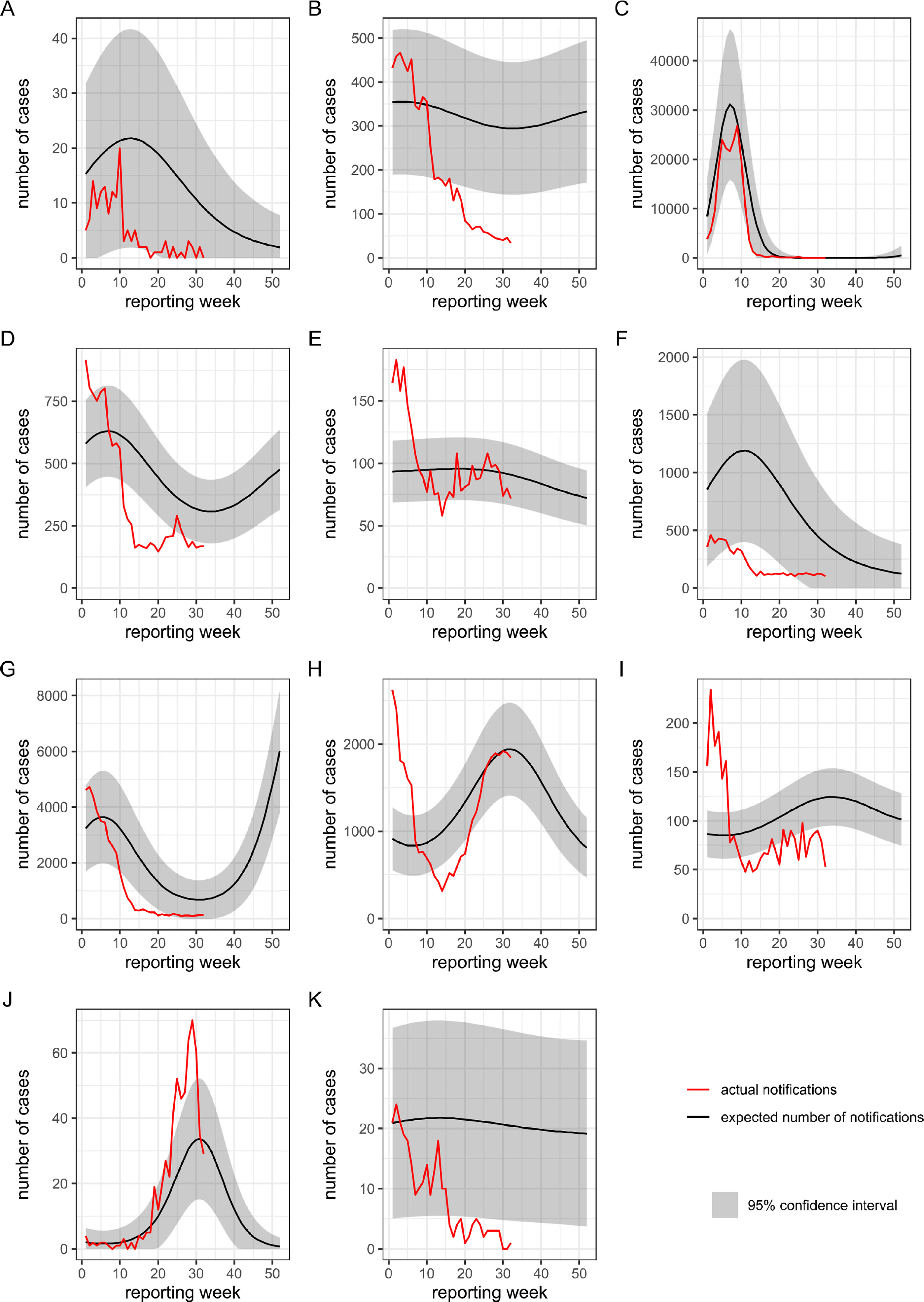

Supplement: Supplementary file 3 [file mmc3.jpg]

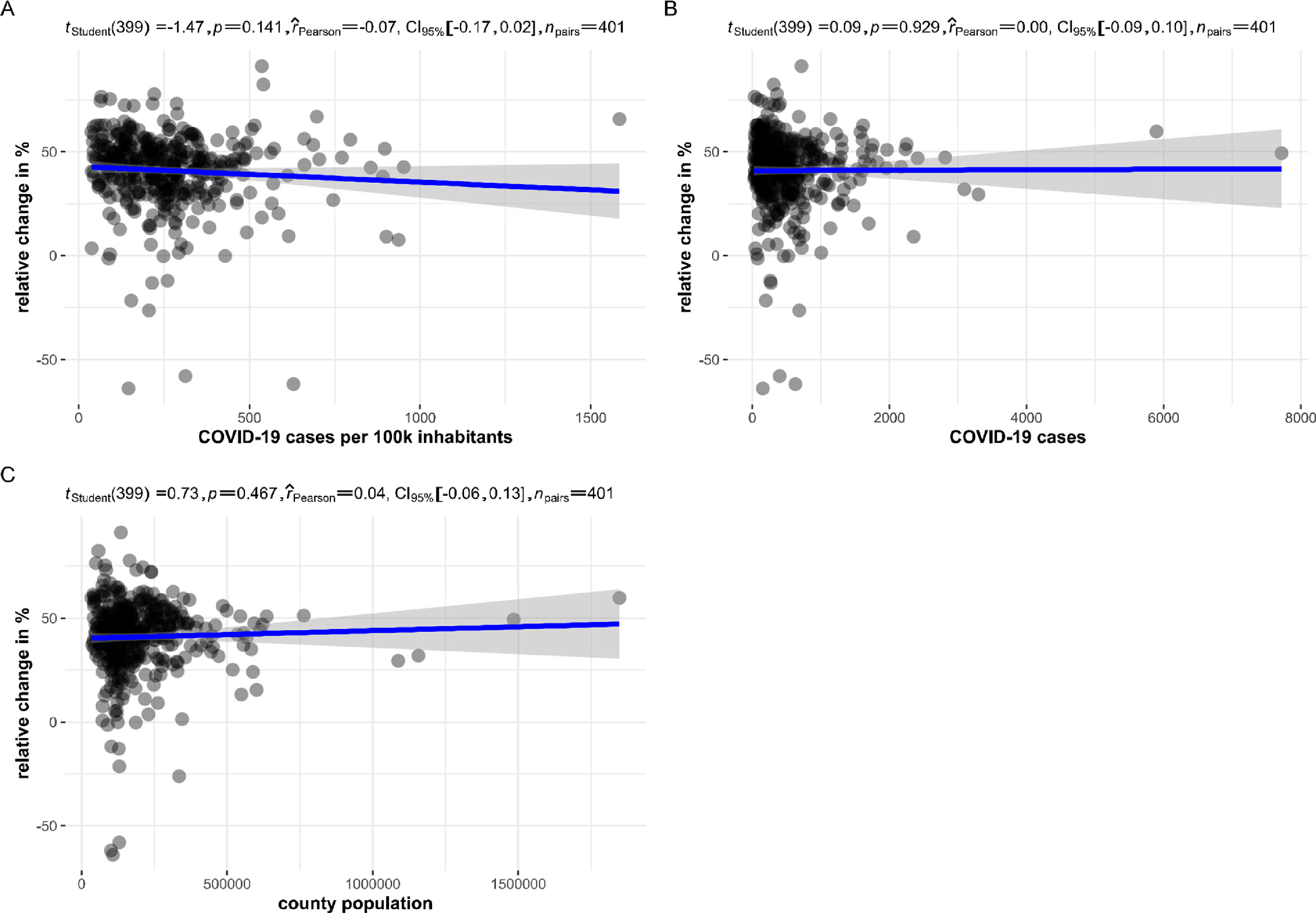

Supplement: Supplementary file 4 [file mmc4.jpg]
