# Supplementary material for: Impact of the COVID-19 pandemic and associated non-pharmaceutical interventions on other notifiable infectious diseases in Germany: An analysis of national surveillance data during week 1–2016 – week 32–2020
Source: Lancet Reg Health Eur. 2021 Jun 19;6:100103. doi: 10.1016/j.lanepe.2021.100103 (PMC8454829; doi:10.1016/j.lanepe.2021.100103)
Supplement: Supplementary file 5 [file mmc5.docx]

**Caption for supplementary material**

*i.e. list of captions for each Supplementary file as they should appear online*

Supplemental Table 1. Overview of included and excluded disease notification categories, by group.

Supplemental Figure 1. Flow chart of inclusion of disease notification categories

Supplemental Figure 2. Correlation between relative change (%) in number of notifications (all notification categories except COVID-19 cases) with A) COVID-19 incidence B) COVID-19 number of notifications and C) population on county level.
